# The interpretation of mu suppression as an index of mirror neuron activity: past, present and future

**DOI:** 10.1098/rsos.160662

**Published:** 2017-03-01

**Authors:** Hannah M. Hobson, Dorothy V. M. Bishop

**Affiliations:** 1 King's College London, London, UK; 2 University of Oxford, Oxford, UK

**Keywords:** electroencephalogram, mirror neurons, mu rhythm, alpha rhythm

## Abstract

Mu suppression studies have been widely used to infer the activity of the human mirror neuron system (MNS) in a number of processes, ranging from action understanding, language, empathy and the development of autism spectrum disorders (ASDs). Although mu suppression is enjoying a resurgence of interest, it has a long history. This review aimed to revisit mu's past, and examine its recent use to investigate MNS involvement in language, social processes and ASDs. Mu suppression studies have largely failed to produce robust evidence for the role of the MNS in these domains. Several key potential shortcomings with the use and interpretation of mu suppression, documented in the older literature and highlighted by more recent reports, are explored here.

## 1. Mu suppression in the past, present and future

**Figure 1.**
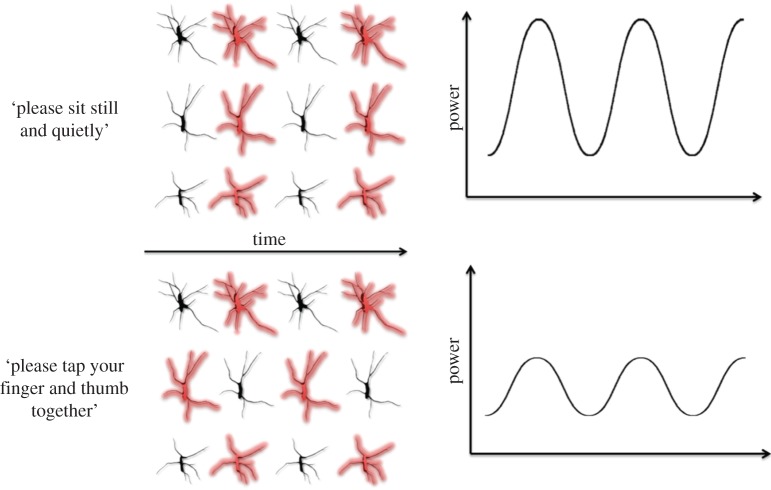
Two conditions—a baseline condition (*a*) and an active condition (*b*)—are represented here. Bold cells represent neurons firing. In the baseline condition, the participant sits motionless. When at rest, the cells in the sensorimotor cortex fire together, leading to higher power in the mu frequency band. In the active condition, the participant is asked to move, generating motor-cortex activity. This leads the sensorimotor cells to fire out of synchrony, leading to reduced mu power. Change in mu power is indexed by subtracting the baseline period from the active period. A negative value (suppression) indicates motor-cortex engagement.

Mu is a rhythm observed in a typical human electroencephalogram (EEG), usually defined as the frequency band 8–13 Hz, thought to arise from the sensorimotor areas. Changes in mu power (i.e. the strength of the mu frequency band) have been used in recent years as a means to study the human mirror neuron system (MNS). Typically, investigations examining these changes take the form of mu suppression studies, in which the power of mu is compared between a baseline condition (which is not expected to excite mirror neuron activity) and an experimental condition (figure 1).

Mu suppression studies have increased in number over the last decade. A recent meta-analysis surveyed 85 studies conducted since 1990 (including data from 1707 participants). This included only studies that examined mu rhythm activity in typical participants, and used an experimental paradigm that had an action observation condition or an action execution condition (or both) [1]. Of these 85 studies, 49 were conducted since 2010. Cuevas and co-workers [2] also noted an increase in the use of mu suppression studies with infants.

The human mu rhythm has a long history, going back to some of the earliest EEG experiments, long before the discovery of mirror neurons. One aim of this review is to give a brief overview of the history of mu, before its reconceptualization as a proxy for MNS engagement. Furthermore, given the widespread and increasing use of mu suppression to explore the existence, reactivity and potential function of the human MNS, it is pertinent to consider whether this method represents a sufficiently reliable and valid technique to infer MNS involvement. Thus, a second aim of this review was to outline problems in contemporary mu suppression studies. Finally, while traditionally mu suppression has been assessed in response to simple action observations, and the primary function of the MNS was regarded as action understanding [3–5], contemporary theories have expanded to posit a role of the MNS in language, social processes and autism spectrum conditions [6–9]. This review will also consider whether mu suppression studies can throw light on these additional putative functions of the MNS.

## 2. Mu suppression: a brief history

### 2.1. Early studies of mu suppression: ‘the rhythm en arceau’

The human mu rhythm was first described by the French scientist Henri Gastaut [10,11], and was termed the rolandic wicket rhythm, or the *rythme rolandique en arceau*, due to the waves' arch-like or wicket-like shape. Early observations were that these waves seemed to arise from the rolandic regions, at a rate of around 7–11 s^−1^ (in contemporary work, the mu frequency band is commonly defined as 8–13 Hz).

In the 1950s, the mu rhythm was thought to be a rare phenomenon, and was even considered indicative of psychopathology. Schnell & Klass [12] identified mu in just 2.9% of their participants. Gastaut *et al*. [13,14] found mu in 14% of a population of 500 healthy male adults, and its occurrence was suggested to be related to psychopathic personality traits. Gastaut & Bert [15] proposed that the cause of mu could be the same cause of psychosomatic traits in neurotic individuals, suggesting ‘*the rhythm ‘en arceau’ is the rhythm of subjects ill at ease in their skin*’ (p. 441). Somewhat later, a link between mu and epilepsy was also suggested, and there were even positive findings concerning the effects of mu biofeedback training and epileptic symptoms [16]. This is reminiscent of recent work linking autism spectrum disorders (ASDs) to mu abnormalities, and attempts to use neurofeedback with this population; see §4.3.

As noted by Niedermeyer & Silva [17], the arrival of standardized caps made it easier to routinely identify mu rhythms from EEG sites C3 and C4 (central sites situated over the sensorimotor cortex), and new techniques for analysing EEG also established that mu occurred more commonly than previously thought. Kuhlman [18] noted that mu activity, with its characteristic wicket shape, was rarely visually identified in the EEGs of their participants, but power spectral analysis revealed it in around half of their sample. By calculating coherence, Schoppenhorst & Brauer [19] were able to identify mu in 60% of 54 participants, a much higher proportion than discovered previously. They also suggested that effects of varying vigilance may contribute to difficulties in identifying mu in some participants.

As it became apparent that mu was not an unusual or particularly pathological phenomenon, new theories emerged about what mu rhythms could be related to. One prominent theory is that mu represents the resting activity in the sensorimotor cortex, and that suppression of this rhythm reflects these regions of the brain becoming active [18,19]. Indeed, in Gastaut's early studies it was recognized that participants' own movements blocked the mu rhythm, and further experiments found that mu could be blocked not only by spontaneous movements but also passive movements, reflex movements and movements to command [20].

Event-related desynchronization or synchronization (ERD/ERS) describes the reduction or increase of a given power band relative to a baseline. Largely, ERD and ERS are thought to reflect cortical activation and idling, respectively. The basic principles of using ERD/ERS in relation to alpha/mu (8–13 Hz) and beta (13–35 Hz) band activity are documented by Pfurtscheller & Lopes da Silva [21]. Using ERD/S, researchers have demonstrated the reactivity of mu to people's own movements, and suggested that there may be different types of mu rhythm, perhaps corresponding to different areas of the motor cortex [22]. However, it is the responsiveness of mu to other forms of stimuli that have generated so much research over the last decade.

### 2.2. Mu suppression and the discovery of mirror neurons

As well as reacting to participants' own movements, mu is suppressed by observing the movements of others. Reports of mu suppression during the observation of movements arose as scientists began to examine EEG responses to film projections. In a delightful paper by Gastaut & Bert [15], the authors describe their observations of their participants' EEG while watching a film reel of a boxing match: ‘*It* [mu] *decreases or disappears completely when the subject changes his position on his seat or when he readjusts his tonus. It also disappears when the subject identifies himself with an active person represented on the screen. This phenomenon is particularly interesting to study during a sequence of film showing a boxing match. A few seconds and, at times, less than a second after the appearance of the boxers all type of rolandic activity disappears in spite of the fact that the subject seems completely relaxed and that there is no noticeable change of posture. The relation between the blocking of the ‘arceau’ rhythm and the image of boxers in action is unquestionable. In the middle of this particular film strip, the camera is suddenly turned from the ring to the spectators in the hall for a few seconds. In many subjects the rhythm ‘en arceau’ reappears during this short period and vanishes again as the boxers reappear on the screen*’ (p. 439).

In the 1980s, a team of Italian neuroscientists identified cells in the macaque brain that fired both when the animal performed an action and when it viewed an action being performed by another [4,5]. These cells were subsequently named mirror neurons, and the observation that the sensorimotor cortex became activated when viewing movement evolved into the mirror neuron theory of action understanding [3]. Following the discovery of mirror neurons in the macaque, the phenomenon of mu suppression took on a new interpretation. The mu band arguably shows similar response properties to mirror neurons. Parallels were drawn between mu and mirror neurons, and a reduction in mu activity was suggested to be a signature of mirror neuron activity [23,24].

Original experiments in monkeys had suggested that mirror neuron activity was related to goal-directed actions specifically; these classic studies used stimuli that showed a hand interacting with an object. In the animal literature, equivalent movements that are not directed to an object do not cause mirror neuron activity [4]. Mu suppression studies with human participants found that stronger mu suppression occurred when viewing another's hand in a precision grip (i.e. a grip that could be used on an object) rather than in a neutral, non-grip position, and that object interaction produced greater mu suppression than conditions without object interaction [23,24]. It has been proposed that this object effect is evidence that mu suppression is related to mirror neuron activity in humans. Arguably, however, a strict interpretation of the animal recording work would suggest that mu suppression should not occur at all when viewing actions that do not relate to an object. Instead, some authors have speculated that MNS responses to non-object-directed actions are a distinctive property of human mirror neurons, and that this difference from other primates may represent a departure from our common ancestors. It is further proposed that this development may have played an important role in the evolution of language [25]. However, a more prosaic explanation is that mu suppression may be measuring the activity of areas downstream from mirror neurons, rather than mirror neuron areas *per se* [24].

As these pioneering studies suggesting that mu suppression could be harnessed for research into the human MNS, many more action-observation experiments have been conducted. This line of inquiry was recently reviewed by Fox *et al*. [1] in a meta-analysis, and the authors concluded that their analysis did show that mu suppression could be used as a proxy for human mirror neuron activity. However, others have contested that mu suppression reflects mirror neuron activity. Coll *et al.* [26] reported that mu suppression indexed sensory mirroring but not motor mirroring, a finding that undermines the important connection between action and perception that mirror neurons are thought to represent. Furthermore, the conclusions by Fox *et al*. [1] were challenged by Hobson & Bishop [27]. We argued that experimental measures of mu suppression were often confounded by non-mirror processes. In our own results mu suppression during action observation was not specific to biological motion, or necessarily specific to the central electrodes situated over the sensorimotor strip. The validity of mu suppression as a measure of the human MNS is thus a current topic of debate.

## 3. Is mu suppression a good measure of the mirror neuron system?

### 3.1. The scientific quality of mu suppression studies

As mu suppression is already widely used in cognitive neuroscience to infer roles for mirroring systems in higher social processes and clinical disorders, the question of whether mu suppression is a good measure of mirror neuron activity is an important one. A recent meta-analysis concluded that while mu suppression offered a valid means to investigate MNS engagement, there were several limitations common in the literature [1]. These problems included issues relatively specific to the field of mu suppression, including the fact that few studies report changes in power at sites other than the central electrodes, making it impossible to be sure that effects were not being driven by changes in power elsewhere. A related problem is that if there are attentional differences between conditions, this could produce widespread changes in another power signal, alpha, that could mimic mu suppression. We echo these recommendations, but also note several other problems in the mu suppression literature, some of which apply also to the wider field of neuroimaging and psychology. We consider these broader points first, before discussing some design issues specific to mu suppression studies.

First, mu suppression studies generally suffer from small sample sizes and consequent low statistical power. In studies that use clinical groups such as autism this is understandable, as these groups can be hard to recruit and may poorly tolerate the EEG procedure. Even with non-clinical samples, however, it has been customary to use sample sizes of 20 or less. It is easy to appreciate that small sample sizes reduce the likelihood of detecting a true effect. However, it is often assumed that if an effect *is* found, even when the sample size is small, then this effect must be true. However, this assumption is inaccurate—a lack of power also means that significant effects are *less likely to reflect a true effect* [28]. In the field of neuroscience, low power is commonplace, increasing the risk of false-positive effects, overestimation of effect sizes and problems reproducing effects in subsequent studies [28]. The number of participants required for a given study depends on a number of factors including analytical design, number of conditions, the expected effect size, correlations between measures and more. Thus, there is no set number of participants that a mu suppression study should include. However, as a rough guide, a repeated-measures design with two factors each containing two levels analysed in a two-way ANOVA would need 40 participants to be sufficiently powered to detect a medium-sized main effect with 90% power.^1^ To detect an interaction, 47 participants would be needed.

Second, mu suppression is a phenomenon with substantial analytic flexibility, and this is another known risk factor for poor reproducibility [31]. For instance, mu suppression studies vary on what frequency band is considered ‘mu’. Frequency bands are not distinctive categories but are flexible ranges that have arisen from the EEG literature, which means that mu suppression papers can employ slightly different frequency bands from each other. The ‘mu band’ has been defined in previous experiments as: 8–12 Hz (e.g. [32]), 8–13 Hz (e.g. [33,34]), 8–15 Hz (e.g. [35]), 8–16 Hz [36], 10–14 Hz [37], or split into bands of upper and lower activity (e.g. [38,39]). Indeed, while many mu suppression experiments define mu as alpha-band (8–13 Hz) activity, mu waves are actually considered to be composed of contributions from two frequencies, including alpha and beta (13–30 Hz), and have characteristic peaks at approximately 10 and approximately 20 Hz. Some research has suggested that beta-band, rather than alpha-band, activity may be a better indicator of MNS engagement (however, see [27]). Thus, some investigations have examined higher and lower mu bands, on the basis that alpha-mu and beta-mu may have different patterns of responses, or examined both alpha and beta activity at the same time. Other researchers have argued that the correct frequency band may have to be calculated from individual to individual, akin to functionally defined sites in magnetic resonance imaging. This could be especially important, as the mu rhythm has been argued to be a target for neurofeedback, and methods for calculating individual frequency bands have been proposed [21]. While there may indeed be a theoretical rationale for splitting the mu rhythm, or selecting a higher or lower or narrower or wider band to examine, it is problematic if these decisions are based on the same EEG data that are to be analysed. This leaves scope for researchers to select a frequency band that provides the best results to fit their hypothesis, introducing circularity into the analysis [40].

Related to the issue of analytic flexibility is that of studies calculating a large number of correlations, or running ANOVAs, without proper correction for multiple testing [41]. These studies are arguably exploratory in design, and need to be considered as such. While ANOVAs effectively correct for the number of levels within a given factor, they do not automatically correct for the number of factors, or the number of potential interactions between factors. For example, a three-way ANOVA is testing for three main effects, three two-way interactions and one three-way interaction. In such an ANOVA, the probability of finding no significant effects at all (if alpha is set to 0.05) is 0.95^7^ = 0.70. Thus, the likelihood of attaining a significant main effect or interaction is 30%. For a four-way ANOVA, this likelihood increases to 54%. ANOVAs are not only problematic in mu suppression literature, but also in the wider EEG field and behavioural sciences [42] (see also the blogpost by Bishop [43] for a discussion of these issues in relation to EEG), and as noted by Luck & Gaspelin [44], these problems are still commonplace even in recently published EEG experiments. Examples of these issues are highlighted particularly in §4.2.1.

### 3.2. The problem of alpha

In mu suppression studies, it can be difficult to ensure that changes in the 8–13 Hz frequency band are arising from sensorimotor areas, due to mirror neuron-related processes, and not from other regions in the brain, or other co-occurring processes. If mirror neuron-related processes are occurring during action observation, these will need to be detected in the context of a myriad of other cognitive and perceptual processes which may obscure their detection, or confound it. Indeed, activity in this frequency band, commonly called alpha-band activity, can be observed at many sites, and changes in it have been implicated in a number of processes [45]. What is alleged to distinguish ‘mu’ from occipital ‘alpha’ is topography and responsivity—while alpha is most prominent at the occipital cortex and reacts to changes in visual stimulation and attention, mu is restricted to electrodes over the sensorimotor areas and responds to participants' own movements. Of course, it is quite possible that during action observation both sensorimotor-related mu suppression and attention-related alpha suppression will occur independently; finding significant occipital alpha suppression does not preclude the possibility that MNS engagement has occurred. Nevertheless, the onus is on the researcher to disentangle mirror neuron activity from other cognitive processes involved in attention and perception.

Perhaps the studies best placed to shine light on this are those that have considered how well mu suppression correlates with other measures purporting to measure the MNS. Such investigations include those that have concurrently taken EEG and functional magnetic resonance imaging (fMRI) recordings, with the view to investigating whether these two measures were in good agreement, and if mu suppression could serve as a cheaper, more accessible way to study the MNS [46–49]. Broadly, the results have been positive—the BOLD responses in brain areas considered to be part of the human MNS (including the inferior parietal lobe, dorsal premotor and primary somatosensory cortex) correlated with mu suppression. Intriguingly, while previous authors had speculated that mu suppression was most likely being generated by Broca's area, a key argument for theories around the MNS and language (see §4.1), Arnstein *et al*.'s [46] findings did not support this notion. However, despite these correlations, authors have warned that their results also suggest that mu suppression may also be reflecting activity from other networks, including regions involved in visuomotor processes that are not part of the MNS [47,49].

Another putative index of MNS activity is transcranial magnetic stimulation (TMS)-induced motor-evoked potentials (MEPs). Lepage *et al.* [50] combined EEG and TMS to investigate the relationship between these two measures. As in previous studies, a significant increase in the MEP amplitude and suppression of mu during action observation, imagination and execution were shown—but there was no correlation between these measures. Other evidence for mu suppression validity comes from magnetoencephalogram (MEG) studies. MEG is considered superior to EEG in its ability to spatially localize sources. In MEG mu-suppression studies, the areas significantly modulated by observation and execution were the sensorimotor cortices, but effects were also found at occipital areas [51,52].

Inadequate separation of mu from alpha could potentially mean that reported changes in mu to certain stimuli are actually changes in alpha, reflecting differences in attention between conditions. For example, Aleksandrov & Tugin [53] measured mu suppression during a range of control conditions, including periods of mental counting, or watching the movement of non-biological objects, conditions in which we would not predict mirror neurons to become active. Yet mu suppression during these conditions was not significantly less than in conditions in which participants observed human actions. Furthermore, their most attentionally demanding tasks appeared to produce the strongest mu suppression, suggesting that mental effort could confound mu studies. Similarly, Perry & Bentin [54] highlight that the significant differences between conditions in their mu suppression study may have been due to differences in attentional demands, rather than differences in the activity of mirror neurons, after finding a comparable pattern of changes in 8–13 Hz power at both occipital and central electrodes. One of the classic markers for alpha is the blocking of eye opening; in one study, eye opening had the biggest effect on mu suppression, greater than any of the experimental conditions [38].

It is well documented that alpha activity is tied to alertness and cognitive effort, and some of the earliest reports noted that this was also the case for mu. Chatrian *et al*. [20,55] reported that mu waves depended on changes of vigilance, and that mental arithmetic [20] or problem solving [56] suppressed the mu rhythm. Schoppenhorst & Brauer [19] state: ‘*While visually evaluating routine EEGs we noted that mu waves were remarkably unstable. On closer observation we found that this ability could be attributed to slight changes in the degree of vigilance, often hardly discernible from changes in the alpha rhythm’* (p. 25). And later: ‘*They are very unstable due to their dependence on the degree of vigilance and can be suppressed both by an increase or a decrease in vigilance*’ (p. 31).

Perry & Bentin [54] cautioned that ‘*mu suppression reports should always include not only experimental effects at the central sites, but also the occipital regions to help fully understand the phenomenon being studied’* (p. 1054). Yet mu suppression studies that have considered the activity at the occipital electrodes have had mixed findings. In their concurrent TMS-EEG study, Lepage *et al*. [50] entered the activity from an electrode over the occipital cortex (Oz) into their analysis and found that 8–13 Hz power at this site was indeed suppressed during conditions when participants observed actions, or imagined themselves performing actions [50]. However, mu suppression studies investigating abnormal mu responses in autistic participants have reported that other than C3, Cz and C4, no other electrodes showed a consistent pattern of suppression, suggesting their pattern of results could not have arisen due to confounding from changes in occipital alpha [57–60].

Related to issues around alpha and attention, the choice of baseline technique in mu suppression experiments could have an important role in whether mu suppression is observed or not. As pointed out by Pfurtscheller & Lopes da Silva [21], ‘*the ERD is measured in the percentage of power relative to the reference interval and therefore it depends on the amount of rhythmic activity in this interval*’ (p. 1847). Baselines of inactivity (where participants receive no stimulation and are asked to sit quietly) may inflate alpha power during the baseline period. Then, when the experimental conditions are subtracted from this period, there is increased likelihood of observing a significant decrease in the 8–13 Hz band, which is interpreted as mu suppression and thus motor cortex or MNS engagement. Potentially, different choices of baseline may contribute to inconsistent findings in the mu suppression literature. While the meta-analysis by Fox *et al*. [1] suggested that baseline did not affect whether or not mu suppression was observed, problems have been highlighted with certain baseline techniques [27].

## 4. Mu suppression beyond action understanding

Having laid out the potential shortcomings of mu suppression, we now explore findings from studies that have used this technique to explore the role of the MNS in a number of different functions. While originally the MNS was proposed to function primarily as a substrate for action understanding, the list of potential roles the MNS could play soon expanded. Language, theory of mind and empathy have all been suggested to have a root in the MNS. If mu suppression is a sufficient measure of the human MNS, and if the MNS is involved in these processes, then one would expect to see mu suppression during tasks that involve language, theory of mind and empathy. Here, the evidence from mu suppression studies for mirror neuron involvement in each of these processes is explored.

We drew sources from Psych Info, using search terms ‘mu suppression’, and ‘language’, ‘social’ or ‘autism’. Additional relevant studies were also drawn from references of published papers read. Included papers were published articles, in English, which used human participants (there have been some published experiments examining mirror neuron function in non-human primates using similar EEG techniques). The focus of this review was on adults, but for studies using autistic participants these samples also contained children. When evaluating papers on mu suppression and the MNS, we found that all papers studied a lower frequency band (around 8–13 Hz), with some additionally considering the beta-band (13–30 Hz), but none had an exclusive focus on beta. We sought studies following the reconceptualization of mu as a proxy for mirror neuron activity, spanning the last decade (since 2007). Thus, this review captures the recent trends in mu suppression usage in experiments motivated by mirror neuron-related theories.

### 4.1. Mu suppression during speech and language tasks

The suggestion that language could have a close relationship to the human MNS has arisen via a number of overlapping and interconnected theories. The first is the motor theory of speech perception [61]. This theory (proposed originally quite independently of and without reference to mirror neurons) argued that producing and perceiving speech relies on the same structures and processes, and that speech is thus perceived by listeners as a series of articulatory gestures or motor commands. While there is superficial parity between the motor theory of speech perception and mirror neurons, language researchers have pointed out that the motor theory of speech perception has long been considered incorrect, and that the revival of this theory following the discovery of mirror neurons has done little to address old problems with the theory [62].

The second theory connecting the MNS and language is an evolutionary one, in which a primitive observation–execution matching system is argued to have supported early communication, followed by the development of a more sophisticated MNS which enabled speech. According to this view, language may have begun in our ancestors as a mimetic gesture-based communicative system [9,63–65]. Broca's area (a key language area and possible homologue for F5, the area of the macaque brain in which mirror neurons were originally discovered) has a central role in the evolutionary MNS--language theory; Rizzolatti & Arbib [9] suggest that Broca's area was originally a region that served action recognition rather than language, and that this was a ‘neural prerequisite’ for the evolution of communication and eventually speech.

A third theory linking the MNS and language is the notion that language is ‘embodied’, and that the existence of the MNS provides evidence for this embodiment. Embodied cognition accounts encompass a number of different domains and processes, including action understanding [66] and executive function [67]. Broadly, these accounts argue that cognition is grounded in perception and action, and true understanding of cognition therefore requires an appreciation for the environment and the resultant perceptual experiences of the organism, and the actions they perform as they move through their world. (Note, however, that embodied cognition theories are very diverse in their claims (see [68]).) Language has been considered from an embodied cognition framework [6,69]. As described by Gallese [6], the embodiment process can be considered at multiple levels—the ‘vehicle level’ (this is essentially the motor theory of speech perception), and the ‘content level’ (the semantic content of words). Thus, this theory encapsulates and expands beyond the motor theory of speech perception—motor resonance is not just for articulatory gestures, but for the content of the sentence itself. For example, hearing or reading the word ‘kick’ may lead to the simulation of ‘kick’ in the hearer's motor cortex.

Having laid out the theoretical links between language and the MNS, what do mu suppression studies suggest about the role of mirror neurons in language and speech processing? Studies examining mu suppression and language have examined both auditory and visual stimuli, and stimuli at the level of phonemes and meaningful sentences. Evidence for mu suppression during speech sounds is considered first, followed by suppression during sentences (which has received less research attention).

Findings of mu suppression during *visual* linguistic stimuli are equivocal. In a MEG study using seven participants, [52] examined mu responses to various oro-facial visual stimuli, including object-less mouth movements, mouth movements directed towards an object (a straw) and linguistic mouth movements. In agreement with previous findings of hand video stimuli, mouth movements directed towards the straw produced the strongest mu suppression. Linguistic mouth movements, however, produced no significant changes in mu power from baseline. The authors of the paper concluded that linguistic stimuli are processed differently from other human movements. However, their results would also suggest that if mu suppression is a measure of mirror neuron activity, mirror neurons have a limited role in the processing of language, when considering visual speech at least.

Other studies have suggested that mu suppression does occur when participants view visual speech stimuli. Crawcour *et al.* [70] investigated mu responses to both visual and auditory speech stimuli. Their 14 participants were presented with nine conditions with different audio-visual pairings, which included visual or auditory speech, visual or auditory noise, and non-biological stimuli (a kaleidoscope pattern or a tone). The only conditions to induce significant mu suppression were those with visual speech stimuli. Conditions in which speech sounds were presented without visual lip movements failed to produce significant mu suppression, suggesting that mu suppression during speech is dependent upon visual stimuli being present. However, it should be noted that this study examined data only from the central electrodes. The extent to which attentional differences between conditions could have driven the effects is thus unclear.

Indeed, task demands may have an important role in determining whether mu suppression is observed to auditory stimuli. Cuellar *et al*. [38] investigated mu suppression during different speech processing tasks, with varying task demands. Unlike previous experiments, their stimuli were auditory only. In the first of their two experiments (using 10 participants), only their most demanding task induced mu suppression, and only at electrode C3. They suggested that this indicated left-lateralized sensorimotor activity when discriminating speech in noise (and only when considering the 10–12 Hz sub-band). In experiment two (with 13 participants), the only significant suppression was found in a task requiring participants to segment the speech stimuli into phonemes, and again only at electrode C3. Collapsing speech versus tone conditions did produce a significant effect that suggested mu suppression is stronger to linguistic stimuli than general auditory stimuli such as tones. A study of 16 participants by Bowers *et al.* [71] found that passive listening to speech sounds did not induce significant mu suppression compared with passive listening to noise, but that active listening (in which participants had to make a judgement about the sounds they heard) did lead to suppression in the beta frequency range (13–30 Hz). These researchers used independent components analysis (ICA)—a technique used to decompose an EEG signal into independent components (see [72] for a review of the use of ICA in EEG), which offers quite a different analytical approach to mu suppression from those usually employed. Using ICA, researchers can investigate the presence of a mu component, and whether its response properties match what would be expected of a MNS involved in language processes. Bowers *et al*. extracted left- and right-sided independent components reflecting sensorimotor activity with distinct spectral peaks at 10 and 20 Hz; these showed slightly different patterns of suppression, at 15–20 Hz on the left, and 15–25 Hz on the right. The observed suppression was referred to as mu suppression by the authors, presumably because of the sensorimotor origins of the components, but in terms of the power changes in frequency, the drop is in the beta rather than the commonly adopted alpha range. The effects of task demand observed in these two studies could be interpreted in two ways; firstly, it could indicate a general effect of attentional demand and alpha confounding. Cuellar *et al*. [38] only examined changes in power at the central electrodes and so, in this study, this remains a possibility. For Bowers *et al*. [71], the use of ICA to investigate the estimated source generators of effects lends more credence to the interpretation that alpha confounding is not driving the effects of this study (though note, with only 32 electrodes, the standardized low resolution brain electromagnetic tomography—or sLORETA—analysis is not considered accurate; see [73]). An alternative interpretation is in line with the account of dorsal and ventral stream processing in speech and language, put forward by Hickok & Poeppel [74]; this argues that auditory-motor integration is served by a dorsal stream, and that this process only contributes to speech processing in more challenging environments. Both Bowers *et al*. and Cuellar *et al*. appeal to this framework, arguing that the selective significant findings in conditions with speech in noise suggest that mu suppression effects reflect the activity of this dorsal stream. However, the frequency bands in which significant effects were observed differ in these two studies—for Bowers *et al*., it was the beta band that produced significant results, while for Cuellar *et al*. it was a sub-band of the alpha band.

One of the key findings in the mu suppression literature on action understanding is that mu suppression is observed both when a participant *performs* a movement and when they *observe* the movement of another. Indeed, it is this pattern of responses that led to mu being suggested as a proxy for mirror neuron engagement in the first place. It is therefore important for researchers looking to use mu suppression to suggest a role of mirror neurons in the motor theory of speech perception to compare mu activity in the perception and production of speech. This is non-trivial, as asking participants to produce speech during an EEG is likely to create movement artefact. However, two studies do suggest that mu suppression occurs during the production and perception of speech.

Tamura *et al*. [36] conducted two experiments with 16 and 11 participants, the first of which examined mu rhythm responses during finger tapping, tongue movements and articulation of speech sounds (with and without vocalization). Mu suppression was seen in all conditions (though it was weakest for articulation with vocalization, possibly due to the high rate of trials rejected for movement artefact), showing that mu responses were not limited to participants' hand actions but could also be demonstrated in speech production. However, there were two key problems with this study. Firstly, the mu band for analysis was defined individually as a 2 Hz window centred at the frequency with the maximum power in the 8–16 Hz range in the baseline interval: this amounts to the kind of ‘double-dipping’ noted earlier [75]. Second, whereas finger tapping gave robust mu suppression at central sites, suppression was less localized for the other activities. Given the difficulties with overt production, the second experiment investigated mu suppression during imagined speech production. Participants initially recorded themselves reading out sentences. Then, while silently reading sentences during an EEG, the participants heard either their pre-recorded selves normally, with delayed feedback, or with noise added. Finally, in one condition, the participants read the sentences in silence (without the recordings of themselves reading). Although Tamura *et al*. [36] argued that their results demonstrated mu suppression during imagined speech production, suppression was not specific either to the central sites or to the stimulation period, and again, the process for selecting the frequency band for analysis from the baseline period would be likely to create some false positives. A further limitation of this study is that production and perception conditions were in two separate studies—a crucial test of mirror neuron functioning would be demonstrating mu suppression both during perception and production of the same stimuli within the same study.

Another study that considered whether production and perception of speech were both related to mu suppression was conducted by Jenson *et al*. [76]. They investigated mu components derived from ICA in 20 participants, and identified a mu component common to conditions requiring imagined speech production, actual production and the perception of speech sounds (during a discrimination task), considering findings from both mu-alpha and mu-beta frequency bands. Their discussion of their findings notes that their results are in accord with attention and cognitive processes, not just motor ones. Nonetheless, this study demonstrates that ICA is an alternative analysis strategy that can be used to investigate the response properties of mu, and is the only investigation that suggests a link between mu in production and perception of speech, within the same set of subjects.

While these studies focused largely on mu's responses to speech, few studies have investigated the role of mirror neuron systems and mu in semantic aspects of language, the ‘content level’ described by Gallese [6]. Van Elk *et al.* [37] investigated EEG responses in 24 participants to written sentences, presented one word at a time, that described either humans or animals performing actions (e.g. ‘the duck swims in the pond’ versus ‘the woman swims in the pond’). They considered several EEG measures, including mu suppression (10–14 Hz), beta suppression (20–30 Hz) and the N400. If motor-cortex activation is related to motor imagery, human actions (being easier to imagine than animal actions) should show stronger effects. Alternatively, if motor-cortex activation is related to the retrieval of lexical-semantic information, they argued that stronger effects should be seen in the animal context. This is because the animal noun limits the range of verbs that could follow, as verbs following animal nouns generally have higher cloze probability (for example, in the sentence ‘the duck swims in the pond’ the probability of the word ‘swims' following ‘duck’ is much higher than it following the word ‘woman’ in ‘the woman swims in the pond’). Considering mu responses alone, stronger mu suppression was observed for the animal sentences. However, this was *not* modulated with the cloze probability of the independent sentences (although both beta and N400 were). The mu responses in this study are not easily interpretable, fitting with neither hypothesis laid out by the study's authors. Beta suppression was found to be greater in animal versus human sentences, and relatedly in higher versus lower cloze probability. As two out of three of the EEG phenomena (beta suppression and the N400) showed patterns in keeping with the lexical-semantic retrieval hypothesis, the authors concluded that motor-cortex engagement in these tasks represents the retrieval of lexical-semantic information.

A study using 30 participants by Moreno *et al.* [33] used mu suppression to examine motor-cortex engagement when hearing either concrete-action sentences (e.g. ‘Now I cut the bread’) or abstract sentences (‘Now, I doubt of the plan’) (sic: grammatical error presumably due to translation of original materials from Spanish). In a separate condition, participants were also shown actions (not exactly the same as those described in the sentences) in video clips. Listening to action sentences and observing human actions was found to result in significantly greater mu suppression than listening to abstract sentences. Approaching these results from an MNS framework, this study would suggest that the role of the motor cortex (and mirror neurons) must be limited to verbs or words that have a motor association or a performable action. Thus, the MNS must only underpin certain subsections of language comprehension, and another system must support our ability to comprehend sentences such as ‘Now, I doubt of the plan’. Alternatively, these findings are in keeping with an associative account, similar to the associative account of mirror neuron development (see [77]). Quite possibly action words become associated with performing an action or viewing that action being performed, due to these words often being said or heard when performing or viewing actions. Hearing these verbs therefore activates the motor cortex via these learned associations, but the activation could be epiphenomenal, rather than playing an active role in verb comprehension.

#### 4.1.1. Summary of mu suppression in language tasks

Mu suppression studies of language have mainly concentrated on speech perception and production, as opposed to semantic understanding. Such studies have returned rather mixed findings; many do report suppression during at least some tasks, but in some cases methodological problems reduce confidence in the findings. Some researchers also have advocated investigating mu suppression in communication disorders, though no studies of mu suppression in language-impaired populations have yet been conducted [78].

Considering the evidence for mu suppression in speech perception (as opposed to language comprehension), suppression seems more likely to occur during tasks that require additional processing, beyond passive listening. Two theories could account for this. Firstly, the recruitment of motor areas only occurs when speech processing is sufficiently demanding. Motor areas are effectively ‘drafted in’ as an additional resource for the task, which would suggest that motor-cortex-based speech perception is not the only means to process speech sounds, and acts as an additional support, rather than a core process in speech perception (see [74,79]). This would represent a much weaker version of MNS--language theories. The second, and arguably more parsimonious theory is that when speech perception (or any) tasks become sufficiently difficult, suppression in the alpha band is seen due to attentional effects. Alpha-band activity occurs all over the head, not just in the sensorimotor cortex, and changes in task engagement and mental activity can lead to suppression of the 8–13 Hz band, potentially quite independent of motor-cortex activity.

Finally, there is also a lack of studies that examine mu suppression in both perception and production within the same experiment, a test that seems crucial given that the crux of the motor theory of speech perception is the overlap between substrates supporting perception and production.

In sum, it is debatable to what extent speech and language tasks lead to mu suppression independent of other potential confounds, or to what extent mu responses to speech stimuli align with what would be expected if the MNS plays a key role in speech and language processes. More confidence in findings could be achieved if some of the key studies could be replicated in a pre-registered design, in which hypothesis, methods and analytical approaches are decided upon prior to examination of the data, to prevent retrospective hypothesis fitting. These theories make clear, testable predictions and would seem well suited to this method of evidence-gathering (for a good guide to replication and pre-registration, see [80]).

### 4.2. Mu suppression in social processes

Following on from the original theories purporting that the MNS underpins action understanding, researchers have used mu suppression techniques to investigate the activation of the MNS in a number of related higher social processes, including empathy and theory of mind. Furthermore, there have been several investigations using mu to assess the role of the MNS in perceiving biological motion. Findings from mu suppression studies examining these skills are reviewed in turn here. Theories that link the MNS to empathy are highly similar to the theories that link the MNS to the separate but related construct of theory of mind. Therefore, these theories will be briefly discussed together, before examining the evidence for mu suppression during these different processes.

#### 4.2.1. Empathy

One theory proposed to explain how we understand and empathize with the thoughts, feelings and beliefs of others is simulation theory. This theory posits that the same mental resources used in our own thinking and emotional responses are also used to understand the thoughts, feelings or beliefs of others, and stands in opposition to ‘theory’ theory—the notion that understanding others draws on empirical knowledge [81]. Related to simulation theory is the perception–action model of empathy [82]. This argues that empathy is based on neural simulation—i.e. seeing others performing actions, or expressing emotions, engages the same neural networks for the execution of action, or the experience of the same emotions, in ourselves. This engagement leads to associated autonomic and somatic responses. We feel what the other person is feeling. The perception–action model could reasonably be considered to be a biological-level account of the cognitive model of simulation theory, and the human MNS could be regarded as the site of perception–action linkage. If the MNS has a role in empathy, then tasks considered to require it or cause it should result in greater mu suppression than tasks that do not.

Evidence for MNS engagement during empathy-related tasks was examined by Moore *et al.* [83]. They measured mu responses in 22 participants while viewing (happy or disgusted) faces, or buildings, after using ICA to extract components corresponding to left- or right-sided mu rhythm components. The viewing of faces was administered under two conditions—in one, participants were asked to empathize with the person they viewed, and in another they were asked to rate how attracted they were to the face. In the buildings condition, participants rated how much they liked the building. Greater suppression was obtained when participants viewed faces as opposed to buildings, although there was no effect of the empathy/non-empathy condition. However, buildings produced significant suppression in 47% of participants (compared with suppression to faces in 76% of participants).

Two theories are posited to explain these results—one is that an empathic response (and a mirror neuron response) to faces is automatic, and thus there is no effect of asking participants to empathize or not, as the activation of the MNS occurs anyway. An alternative hypothesis that the authors concede is that as no facial electromyogram (EMG) was recorded, participants might automatically (and unconsciously) mimic the facial expressions of the faces they view. Participants' own facial movements would lead to motor-cortex engagement, and thus mu suppression. The finding of significant mu suppression to buildings is argued to need more research. This is not predicted by any theory and raises the possibility that the interest generated by the stimuli, rather than empathy, may have been a factor in causing suppression.

Perry *et al.* [32] examined mu responses to viewing painful or non-painful stimuli. Twenty-eight participants viewed pictures of ‘painful’ (a hand being pricked with a needle) or ‘non-painful’ (a hand being touched by a cotton bud) stimuli. They were also told that some of these pictures were of a patient with a rare disease in which the needle would not cause pain, but the cotton bud would. Significant suppression of alpha-band activity was seen to painful stimuli—including when participants viewed cotton buds touching the purported patient's hands (demonstrating empathy). Strongest suppression for stimuli arose from the occipital rather than the central regions, but the authors argue that the actual pain effect (stronger suppression to painful versus non-painful stimuli) arises from the central and frontal regions, rather than the occipital; however, they failed to achieve a significant site by pain interaction. It should also be noted that the researchers used a five-way ANOVA; in the absence of prior prediction, the probability of obtaining at least one conventionally significant (i.e. *p* < 0.05) result from the 29 main effects or interactions in this analysis would be 0.77, i.e. 1 − 0.95^29^ [42].

The studies detailed so far consider average mu responses to different conditions, requiring or not requiring empathy. A different line of inquiry has examined individual differences in empathy and MNS activity, using mu suppression as a measure of MNS engagement. Indeed, some studies have suggested that female participants exhibit stronger mu suppression than males, a difference thought to be related to gender differences in empathy, and also to complement the extreme male brain theory of autism [84,85]; however, the meta-analysis by Fox *et al*. [1] suggested an effect in the opposite direction, and Hobson & Bishop [27] found no significant gender effects on mu suppression. Furthermore, several studies have also investigated correlations between individual mu suppression, personality traits, and self-reported social and empathy skills. Cheng *et al.* [85] noted a positive correlation between mu suppression and the personal distress subscale of the interpersonal reactivity index (IRI), and suggest that ‘*the EEG mu rhythm can be a potential biomarker of empathic mimicry*’ (p. 4). They also noted a negative correlation between mu suppression and the systemizing quotient (SQ), a dimension related to the extreme male brain theory of autism. Unfortunately, the meaningfulness of these correlations is questionable. First, the IRI has four subscales, and the authors also report using the empathizing quotient, SQ and emotional contagion scale. Thus, there were seven measures which were investigated for a correlation with mu suppression, but the authors do not report any corrections made for multiple tests. Furthermore, the effect sizes of these statistically significant correlations are small. Across their 40 participants, the correlation between the SQ and mu suppression was −0.124, and for the personal distress scale it was 0.118.

Other studies have found moderate relationships between mu responses and questionnaire responses. In a later MEG study, this group considered mu suppression to viewing painful versus non-painful stimuli, and found a correlation between mu suppression and the perspective-taking subscale of the IRI of 0.36 and 0.37 (the correlations were given separately for the right and left hemispheres, respectively) [86]. Woodruff *et al.* [35] investigated the relationship between mu suppression and self–other discrimination, a key component of contemporary theories about empathy and perspective taking. In their sample of 39 participants, they found a moderate correlation between the perspective-taking component of the IRI, and the *difference* between mu power between execution and observation conditions. The greater the difference, the higher is the score on the questionnaire (*r* = −0.36).

Yet other studies have reported failing to find correlations between mu suppression and measures of empathy [83,87,88]. The latter study found a significant correlation between mu suppression and empathy in the *opposite* of the predicted direction. When Silas *et al*. [87] investigated the associations between socio-emotional scales, mu suppression and gender in their sample of 33 participants, they did find that mu suppression was stronger in females, and that females scored higher on self-report socio-emotional questionnaires—but there were no correlations between individual differences and mu suppression. They suggest that while the sex difference in mu suppression may be real, it is unrelated to socio-cognitive abilities.

Leading on from work on empathy, social scientists have also considered how mu suppression may be used to study the neural mechanisms for intergroup relations and prejudice. Drawing on the perception–action model of empathy, Gutsell *et al*. [89] hypothesized that individuals with more prejudice would show reduced mu suppression to the outgroup: ‘*These* [intergroup] *biases… might be a manifestation of a more basic and general bias: perception–action-coupling for gross motor responses—the physiological process thought to be at the core of interpersonal sensitivity—might be impaired in response to disliked outgroups. Such a fundamental bias, would not only make it difficult to empathize with outgroup members' suffering, but also to understand their actions and intentions, potentially hampering smooth intergroup interactions and communication’* (p. 842). In a sample of 30 Caucasians, Gutsell *et al*. [89] found significant differences between the mu suppression towards in-group versus out-group members, and significant correlations between prejudice scores and mu suppression towards out-groups. The correlation they report is moderately large (*r* = 0.52). Gutsell & Inzlicht [90] discuss further research that followed on from these findings, which suggests that mu-suppression biases can be modified by engaging participants in a perspective-taking task, and that mu-suppression biases correlate with beliefs about genetic overlap between different racial groups. Correlations on small samples have wide confidence intervals and one needs to be cautious about interpretation, especially given variation from study to study. Furthermore, it seems quite plausible that viewing in-group and out-group members could have differential attentional effects, as in-group members may therefore be more likely to engage our attention, suppressing alpha (rather than mu). There is some tentative support for a link between mu suppression and empathy but findings need replicating in a pre-registered study.

#### 4.2.2. Theory of mind

Despite considerable amount of research on empathy and mu suppression, only one study was found that used mu suppression to investigate MNS involvement in theory of mind. Pineda & Hecht [91] argued that their mu suppression study of 23 participants provided evidence of a dissociation of different theory of mind routes. They appealed to a theory of mind framework by Tager-Flusberg & Sullivan [92], which suggests that theory of mind could be considered as having socio-cognitive and socio-perceptive components. (One could broadly link the socio-perceptive component to the simulation account of theory of mind outlined earlier, while the socio-cognitive account may be thought of as similar to the ‘theory’ theory of mind approach.)

Pineda & Hecht [91] employed tasks argued to measure these different socio-perceptive and socio-cognitive components. To measure socio-perceptive processes, they used a task that required participants to match images of eyes, based on the eyes' emotion, race or gender (the latter two acting as control tasks). For the socio-cognitive processes, they used a cartoon task, in which participants guessed the last panel of a comic strip. The comics require either mental attribution (understanding what the person is intending to do), or an understanding of physical causality. With regard to the physical causality comics, some contained characters, but intention reading was not required (e.g. seeing someone's scarf blown off by the wind), while others contained no characters at all (e.g. seeing a bomb explode). The authors argue that their results supported a distinction between socio-cognitive and socio-perceptive tasks, and that the MNS is more involved in socio-perceptual than in socio-cognitive tasks. This would be in keeping with the notion that the MNS underlies a simulation mechanism that allows us to experience and understand others' minds.

However, the results of this study are difficult to interpret. A direct comparison of the strength of mu suppression in the socio-cognitive and socio-perceptive tasks is not reported—so it is not possible to say whether socio-perceptive tasks result in greater mu suppression. Furthermore, the pattern of suppression across the tasks does not clearly demonstrate a difference between socio-cognitive and socio-perceptive tasks. For example, while significant suppression was seen during the emotion-matching task, significantly stronger suppression was seen during the race-matching task (though the authors interpret this as showing mirror neuron activity while participants make these race judgements). The authors also argue that the task with comics depicting physical causality with characters is actually a socio-perceptive task, as there are correlations between this and the emotion eye-matching task. Yet it is difficult to see why this physical-causality comic task would be underpinned by the MNS, yet the intention-reading comic task would not be. The key difference between these two tasks was the need to infer intention. This would seem, at face value, to be exactly the kind of skill the MNS was originally proposed to underlie. Pineda & Hecht [91] argue that the intention-reading task is more of a ‘theory’ theory task, requiring empirical knowledge and social cognition, while the physical-causality character and emotion eye-matching task are socio-perceptive tasks, resulting in MNS activation. However, the distinction between these two types of comics (with one being called a socio-cognitive task and the other a socio-perceptive task) seems arbitrary (especially considering the authors conclude that probably both routes are active in both tasks, and mu suppression occurs in all of them).

#### 4.2.3. Biological motion

Several studies have considered mu responses to biological point-light displays. These displays are image sequences created by marking the limb movements of moving bodies with lights. These stimuli offer a solution to the problems of presenting well-matched stimuli to investigate mu responses—social versus non-social stimuli typically differ on a number of basic perceptual factors, while point-light displays allow for a tighter control over such variables.

Mu suppression to these displays has been used to argue that mirror neurons are involved in the processing of biological motion. In a study of 20 participants, Ulloa & Pineda [93] found significant mu suppression to biological point-light displays, but not scrambled motion displays. They argued that their effects were not due to attentional differences, as performance on a continuous performance task did not differ between these conditions—however, no results are reported for regions outside the central electrodes.

Indeed, other authors examining mu suppression to point-light displays have warned about potential confounding effects from occipital alpha and attentional differences in the different conditions. Perry *et al.* [88] examined participants' ability to recognize the different dimensions represented in the point-light displays (emotion portrayed, gender of the model, direction of walking and direction of rolling for the non-biological point-light displays). Participants were slower and sometimes less accurate to make decisions about some of the social dimensions represented in the displays (emotion, gender, intention) than direction of rolling in the non-biological motion condition, suggesting that these tasks were not matched for task difficulty. Furthermore, in the analysis of EEG data of 24 of their participants, they reported results from the occipital regions which showed significant alpha suppression across the conditions, and a pattern of suppression similar to that found at the central sites. Possibly, the authors suggest, biological point-light displays may attract more attention, as these factors have higher ecological value (e.g. needing to know whether someone is walking towards or away from you).

#### 4.2.4. Summary of mu suppression in social processes

The findings so far relating mu suppression to social processes are varied. Studies attempting to use mu suppression as an individual differences measure of the MNS seem particularly problematic. Few strong correlations have been observed (almost none that survive corrections for multiple comparisons), and it is unclear whether other theories not pertaining to mirror neurons could account for some of the findings. Certainly, many studies do not sufficiently consider the potential confounds of alpha or attentional effects in their designs ([88] is a notable exception that clearly demonstrates the importance of considering such issues). Another consideration is whether participants' own movement could confound the effects. Participants generating their own movement would obviously lead to motor-cortex engagement and mu suppression. Therefore, if conditions are able to vary in the amount of movements participants performed, these differences could confound the results of mu suppression studies. Instructing participants not to move may not always be enough, as there is evidence that individuals may mimic others without awareness; automatic mimicry is a phenomenon in which individuals unconsciously mimic the actions or postures they perceive in others. Indeed, automatic mimicry could feasibly mediate reported relationships between empathy mu suppression as there is evidence that participants that score high on empathy mimic more than participants that score low [94]. Across the field, many mu suppression studies do record EMGs from participants, in order to discard trials in which participants move, but this may be a particular issue for mu suppression and empathy research, as automatic mimicry was little discussed in these papers. Overall, there seems in many of these studies no need to appeal to a mirror neuron theory account—alternative accounts, including attentional differences between social stimuli and automatic mimicry effects, could explain these results just as well.

### 4.3. Mu suppression studies of autism spectrum disorders

In parallel to work on mu suppression and empathy and language, mu suppression has also been used to examine the functionality of the MNS in ASDs. Theories that self–other representations may be impaired in autism arose independently of mirror neurons—Rogers & Pennington [95] suggested that such impaired representations could account for the reported imitation difficulties seen in this group, and broader social and communication problems. Most recent reviews have argued that the well-documented imitation difficulties in ASD could be due to an abnormality in the MNS [96], and following suggestions that mu suppression could represent a proxy measure for mirror neuron activity, it was quite logical to use mu suppression set-ups in samples of individuals with autism. Indeed, mu suppression set-ups are likely to be better tolerated by individuals on the spectrum than other imaging methods, such as fMRI.

It must be noted that while autism is certainly the condition most investigated using mu suppression, an outstanding question is to what extent mu suppression deficits are *specific* to autism. Studies so far have also found reduced mu suppression to biological motion in Down syndrome [97] and first-episode psychosis [98]. Finding mu suppression deficits across a range of disorders could suggest that atypical mu suppression responses are reflective of quite general abnormalities in brain development, rather than specifically an abnormality in the MNS.

To date, we are aware of nine studies comparing groups of individuals with an ASD to typical control groups. These are summarized in table 1. There have also been investigations into the benefits of mu neurofeedback training, which have argued that this may represent a potential treatment for autism in the future [103–107]. At present, mu suppression findings with autistic groups have been decidedly varied, with half of the studies concluding that mu suppression during observations of actions is deficient in autism (suggesting abnormal or impaired mirror neuron systems), and half finding mu suppression comparable with controls. There have been some attempts to explain these varied findings by appealing to additional factors; for example, Oberman *et al*. [60] found that mu suppression in their autistic sample was modulated by familiarity with the model (arguably, one could link the findings of Gutsell *et al*. [89] relating mu suppression to prejudice to those of Oberman *et al*. [60], as presumably in-group members are much more familiar with their own group).

**Table 1.** Findings from mu suppression studies with participants with ASD. OM, own movement; BB, bouncing balls; WN, visual white noise; CPT, continuous performance task; ASD, autism spectrum disorder; HFA, high functioning autism.

paper	sample	stimuli/conditions	findings
Oberman *et al*. [59]	10 ASD, 10 TD (age- and gender-matched). Aged 6–47 years.	OM; watching video of hand action (opening and closing hand, same as OM condition); watching video of two BB; WN (baseline).	TDs showed significant mu suppression to OM and observed movements. ASD group showed significant mu suppression during OM only. The lack of suppression in the ASD group was not due to differences in baseline mu power. Neither group showed significant suppression from baseline during the non-biological motion.
		Included CPT—100% accuracy, so inferred that differences between groups are not due to differences in attention.	
Oberman *et al*. [60]	13 TD and 13 ASD, aged 8–12 years.	Stranger opening and closing hand; familiar (sibling/parent) opening and closing hand; video of own hand movement; BB (baseline).	TD group showed significant suppression to all 3 biological videos. ASD group showed significant mu suppression for videos of familiar hands and their own hands only. So the paper's authors inferred that differences between groups are not due to differences in attention.
		Included CPT (100% accuracy).	
Raymaekers *et al.* [99]	20 HFA, 19 TDs, 8–13 years.	OM (finger tapping); observing moving hand; BB; WN (baseline).	Same pattern of findings for both TDs and ASD and no significant differences between groups. ASD groups show intact mu suppression to hand videos, and no significant suppression to BB.
		Included CPT (100% accuracy).	Significant correlation between intelligence and mu suppression, but not between symptom severity and mu suppression.
Bernier *et al*. [57]	14 ASD and 15 TDs, aged 19–43 years.	Rest condition (baseline); observed hand action with object (gripping a manipulandum); execute action (gripping manipulandum to command); imitate action (gripping manipulandum in imitation of videos).	For the ASD group, the observe condition showed significantly less attenuation than execute and imitate conditions. Imitate and execute did not differ. For TDs, there was no significant differences between execute and imitate, or execute and observe conditions. The groups differed on mu suppression for the observe condition—the ASD group showed significantly less mu suppression to TDs. Mu suppression in the observe condition was correlated with imitation skills.
Bernier *et al*. [58]	19 ASD, 19 TDs, aged around 6 years.	Rest condition (baseline); observed hand action with object (gripping a manipulandum); OM (gripping manipulandum to command). Trials in which children did not attend screen were discarded.	Both groups showed mu suppression during OM and observation of hand actions. No correlation with IQ, or communication impairments in ASC. Correlation between imitation of face expression and mu suppression during the observation condition. A subset of children who did not show mu suppression to the observation condition (5 TD and 2 ASD) had poor face imitation abilities.
Ruysschaert *et al*. [100]	18 ASD and 19 TD, aged 24–48 months.	Object observation condition (dangling object); action observation (goal-directed action); action imitation condition (children encouraged to imitate); observing hand movement condition (experimenter did hand actions similar to action observation but with no object).	Imitation score was comparable between groups. Significant mu suppression during hand movement observation, action observation and action imitation task for both groups. No group differences in mu suppression. No correlations with SCQ in the ASD group, moderate correlation in TDs. No correlation with quality of behavioural imitation.
Martineau *et al.* [101]	14 ASD and 14 TDs, aged 5–7 years.	Rest; static scene viewing (e.g. lake); scenes with motion (e.g. waterfall); video of woman performing movements with her legs.	Showed desynchronization of the EEG in the motor cortex and the frontal and temporal areas during observation of human actions. No desynchronization found in autistic children. Note that while commonly cited, this study mainly reports effects for the *theta* band rather than the *alpha* band.
Fan *et al*. [102]	20 ASD and 20 TDS, aged 10–26 years.	Manipulating chess piece (OM); observation of hand interacting chess piece; moving white dot; static cross rest condition (baseline). Included eye tracking.	No visual attention (as measured by fixation) differences found. Participants with ASD failed to imitate the observed actions, but mu suppression was not different from that seen in TDs. Mu suppression during observation was associated with the communication competence of individuals with ASD.
Dumas *et al*. [39]	10 ASD, 30 TD, aged 20–41 years.	Observation of meaningless hand gestures; execution of meaningless hand gestures (not to imitation); spontaneous imitation (subjects were told that they could at will either produce hand gestures of their own or imitate the others’ hand gestures); video imitation (where participants imitated the videos); rest condition.	Analysis of the large mu band showed significant mu suppression for TDs and ASDs for action execution. Only TDs showed significant mu suppression during observation of hand gestures. Considering the whole scalp in the observation condition, TD participants showed a significant suppression of the 8–13 Hz band over the whole scalp, more strongly over the occipital parietal region. ASDs did not show such significant alpha suppression. For upper band only, significant differences for the frontal and occipito-parietal region—greater suppression over the OP region in the TD group, and an increase of alpha in the frontal region in the ASD group.
		Whole brain approach, considered different mu bands (large mu band: 8–13, and 2 narrower bands: 8–10, 11–13).	

However, the most recent paper to investigate mu suppression abnormalities in autism points towards abnormalities in the mu frequency band, but suggests that these abnormalities arise from areas not usually associated with mu, but rather with alpha. When only examining the central electrodes, such as is typically done in mu suppression experiments, Dumas *et al.* [39] replicated previous reports of reduced suppression to actions with objects. However, when considering differences across the whole scalp, Dumas *et al*. [39] found abnormalities in the alpha frequency band in the frontal and occipital regions in their participants with ASD. Indeed, there is evidence that the broader alpha band, as opposed to mu, is abnormal in ASD; Mathewson *et al*. [108] noted in their study that participants in the ASD group had greater alpha power in an eyes-open condition, and that they showed smaller occipital alpha suppression when comparing eyes-open to eyes-closed conditions than typical controls. Reduced suppression in the alpha band is therefore not specific to mu regions or biological stimuli.

Furthermore, it is plausible that attention may be different between ASD and typical participants when viewing biological motion, and that this could be reflected in differences in alpha activity. Attention to social stimuli has been shown to be abnormal in ASD (see [109] and [110], for examples and discussion of these issues in both auditory and visual domains, respectively). Previous mu suppression reports argued that attentional differences could not account for mu suppression findings in autism, because behaviourally measured attention (usually measured though continuous performance tasks) showed no differences between groups. However, these tasks represent rather gross measures of task engagement and may not be picking up on more subtle attentional differences between groups.

Potentially even the positive neurofeedback training findings could be, at least in part, explained using attentional theories. Both studies in a report by Pineda *et al.* [111] found that the experimental group showed improvements in measures of attention deficit hyperactivity disorder symptoms and sustained attention ability. No differences in improvements in imitation skills were found when comparing placebo and experimental groups, and improvements in communication, perception and sociability were inconsistent between the two studies. This suggests that mu suppression neurofeedback training may have had more of an effect on general attention and alpha than on the MNS and the skills it has been suggested to underpin.

If the ‘broken mirror hypothesis’ is true, and mu suppression represents a good measure of mirror neuron activity, then correlations and associations between mu suppression and behavioural measures supposedly supported by the MNS and impaired in autism should be evident. Imitation skills are particularly important in this theory—they are a key link between the original cognitive theories about self–other representation in autism and MNS theories now. One suggestion has been that mu suppression abnormalities pertain more to variation in imitation ability than to autism *per se*. Associations between imitation and mu suppression have been reported [57,58] but these have not been well replicated. Ruysschaert *et al.* [100] found no association between the quality of children's imitation and mu suppression. Fan *et al.* [102] noted that their ASD group had poor imitation skills and yet showed intact mu suppression. Following neurofeedback training with mu, there were no differences between the improvements in imitation between the placebo or experimental groups [104].

One final comment on mu suppression studies of ASD is on the analyses of these studies. Few of these studies seemed to correct for multiple comparisons in their analyses. Furthermore, of the studies that have analysed their data using ANOVA techniques, *none* have found a significant group effect, nor a significant group by condition interaction [57,58,60,99,100,102]. Thus, it is questionable whether a selective abnormality in mu suppression to observing others is borne out by current data.

#### 4.3.1. Summary on mu suppression studies of autism spectrum disorders

In sum, the evidence for a specific lack of mu suppression to observing human actions in autism is poor. Rather, the collective evidence suggests there may be differences in the alpha frequency band that are not related to MNS dysfunction. As yet, there is no evidence that mu suppression abnormalities are truly specific to ASD, and robust findings linking mu suppression in autism to imitation skills, a behaviour the MNS has been purported to underpin, are lacking.

## 5. Mu suppression in the future: improving mu suppression studies

This review had three aims—to consider the history of mu research, consider whether this method represents a sufficiently reliable and valid technique for inferring MNS involvement in a variety of tasks and settings, and to consider what mu suppression studies have so far suggested about the role of the MNS in its more recently suggested functions, namely language, higher social processes and the development of autism spectrum conditions. Mu suppression started out as a measure of motor-cortex engagement but has been reinterpreted as a proxy for mirror neuron activity. Theoretically, mu suppression could be a comparably cheap, tolerable and simple way to examine mirroring systems in the human brain, and these set-ups have been used with both individuals with autism and young children, populations for which imaging with other methods may not be practical. Mu suppression has been explored in language tasks, in its association with empathy and social processes, and in autism spectrum conditions. All of these domains have been linked to the human MNS, but mu suppression studies provide limited support for MNS involvement in these processes, due to widespread methodological issues with many mu suppression experiments.

As outlined above, there are many reasons to be cautious of mu suppression findings. We are certainly not the first to point out the potential pitfalls of confounding from alpha and attentional changes, but the problem of unaccounted alpha confounding is one that appears across fields of mu suppression studies, including language, social and autism-related research. Our recent investigation into mu suppression baseline techniques has highlighted the potential for alpha confounding when studies use resting baselines, or baseline conditions rather that baseline windows within given trials—of the studies reviewed here, the majority used blocked baseline conditions. Furthermore, mu suppression as a field is seriously underpowered, and analyses commonly include a large number of factors without adequate corrections. Clearly, some effects are predicted by theory, but when there is no clear distinction between hypothesis-testing and exploratory analyses, it can be difficult to evaluate the findings. Future studies will need to be much more robust if important discoveries can be expected to be made about the MNS using this method. Limited sample size is particularly problematic if the goal is to study individual differences [112]. Table 2 summarizes some of the key considerations researchers wishing to use mu suppression should account for.

**Table 2.** Summary of the key points concerning what features mu suppression experiments should aspire to have.

key considerations for future mu suppression experiments
— Careful consideration of baseline for calculation (complete reliance on long resting baseline condition should be avoided).
— Suitable control conditions for attentional demands of the experimental condition.
— Own movement conditions to test linked production and perception, a key characteristic of the mirror neuron system.
— EMG, or means of monitoring participants' own movements.
— Examination of alpha-band changes outside the central electrodes.
— Robust corrections for multiple comparisons.
— Sufficient power and appropriate sample sizes.

In addition to methodological improvements and better scientific rigour, mu experiments may also benefit from different analytic approaches. For example, a small number of experiments reviewed here used ICA. Using ICA in mu experiments has several potential advantages, but also some issues will need to be considered. A few experiments have used ICA to extract mu-related components, which may help to address the issue of alpha confounding—these analyses are no longer based in channel space but rather in component space. Previously, group-based studies using ICA were challenging, because components identified by ICA may differ from participant to participant. Group-wide components-based analyses are now however available. As noted in the study by Jenson *et al*., researchers wishing to use ICA in production-perception designs will need to consider the effect of movement on their ICA. Own movement conditions are important features of mu suppression experiments, because they provide positive confirmation of motor-cortex engagement, and as a key characteristic of the MNS is its joint involvement in motor and perceptual processes, experiments should demonstrate mu changes in both these conditions. However, in Jenson *et al*., power in mu components was reduced in their speech production condition, contrary to predictions that these conditions would result in the strongest motor-cortex engagement. The authors suggest that this was due to the greater proportion of EEG variance that had to be accounted for by EMG activity, and as such caution that researchers using such ICA approaches should not draw conclusions about the strength of engagement of the motor cortex in such production tasks, as this may be misleading. It is also worth noting that, of the experiments reviewed here, ICA did not ensure that results were in keeping with MNS theory; for example, Moore *et al*. [83] found significant suppression to buildings. Relatedly, event-related spectral perturbation (ERSP) analyses were used in a few studies reviewed here. ERSP is described as ‘generalized’ ERD [113], and the method of using an immediately preceding period as a baseline (such as that using in [27]) is logically similar to the approach in ERSP. ERSP analysis functions implemented in statistical toolboxes such as EEGlab offer many options for methods of frequency decomposition, and it is as yet not clear which is the optimal one for demonstrating mu suppression. It would be helpful to have studies that systematically compared different analytic approaches using data that gave robust mu suppression (e.g. during movement) to establish a standard method, as the range of possibilities currently available gives many researcher degrees of freedom, and this hinders reproducible research.

We believe the method of mu suppression can be refined, but it is also worth noting that it is not the sole technique that has been proposed as a means to study the properties of the human mirror system. Firstly, some have suggested that beta, rather than mu, may be an index of MNS engagement [51,114,115], and, in a few cases in this review, positive effects were found for beta-band activity but not for the alpha band (e.g. [37,71]). However, in our own study, we did not find beta effects that were compatible with our *a priori* characteristics of MNS activity [27]. TMS has also been used to suggest the existence of a human mirroring system, and that such systems may be important in speech perception and language comprehension [116,117]. This method is also not without controversy; for example, the mirroring properties observed during these TMS studies have been shown to be altered after relatively short periods of training [118]. An alternative and novel experimental paradigm is repetition suppression. Repetition suppression is widely used in fMRI, but has recently been used in cross-modal experiments to test the responses of mu rhythms [119].

We have focused our review largely on data collected from adults in mu suppression studies (with the exception of some of the studies of ASD); however, researchers have also used mu suppression studies with infant populations to try and address questions about the development of mirroring systems. Other researchers have reviewed mu suppression with infant populations, so this literature has not been re-examined here [2]. However, it is worth noting that the review by Cuevas *et al*. [2] outlines several pertinent problems in many infant mu studies, including the issues of baseline selection and examining changes outside just the sensorimotor regions. They too highlight the need for researchers to consider changes in power at the occipital region, and point out that topographic maps of power distributions across the scalp provided by some infant mu researchers would actually seem to show suppression at the occipital sites. Broadly, the content of infant mu suppression studies has largely been around the processing of others' actions (arguably the traditional remit of mirror neuron theories), rather than broader functions in language and social processes. Work so far has largely concluded that these infant mu rhythms show the same patterns of reactivity to participants' own movement and action observation as the adult mu rhythm, and that mu suppression may represent a means to investigate mirror neuron systems in young children [120–122]. As is apparent from our review, using mu suppression to examine language and social processes in adults has produced few robust findings, and so translating these studies for use with infants, where even less is known about the interpretation of EEG, would seem unwise at present.

Furthermore, a comment not so much on the methodology of mu suppression studies but on their interpretation in wider social cognitive neuroscience: the impression one gets when reading the mu suppression literature is that theories about the function of the human MNS are sufficiently flexible to fit around whatever mu suppression results are obtained. Of course, given that theories about the MNS evolved and developed, we can expect to see mu suppression to stimuli beyond simple hands interacting with objects. But mu suppression has been demonstrated to viewing static buildings, sheet music and Rorschach ink blots [83,105,123]. These are a far cry from the original stimuli used to investigate action understanding. Mu suppression as a field seems to be attempting to simultaneously validate mu responsivity as indexing mirror neuron activity, *and* use this responsivity to suggest what stimuli the MNS is responsive to, but this logic is circular. Ideally, the field needs to agree what to expect the human MNS to respond to, examine whether mu suppression meets these expectations and reject it as a measure of the MNS if it does not meet them. Recent work on mu suppression suggests we need far more work establishing the reliability and validity of our measures, and agreeing on appropriate analysis pipelines, before we can use this approach with confidence to index activity of the human MNS [27].

While new data will be useful in making progress, this review also sought to reach back for evidence. Mu changes have long been considered to index motor-cortex engagement, well before mirror neurons exploded into the field of cognitive neuroscience. Considering mu's history, and how mu studies have changed over the last decade, should lead to reflection on how mu suppression should be conducted in the future. We hope that researchers will use this synthesis of the evidence to design and implement careful and considered mu suppression experiments in the future that can successfully rule out the confounds we and previous authors have outlined.

## Footnotes

1 This was calculated using G*Power [29]. 90% power is the minimum accepted by most journals offering pre-registration. A conservative estimate of non-sphericity correction was employed, and indeed it is typical for this assumption not to be met. We balanced this conservativism by entering a relatively high correlation between the measures, 0.7, higher than that reported by the mu suppression study of Tangwiriyasakul *et al.* [30]. Lowering this correlation would increase the number of participants needed, and relaxing the non-sphericity correction would reduce the number of participants needed.
